# Deepening the Understanding of Platelet‐Derived RNA as Biomarker for Glioblastoma Through GSEA, TDEA, and Elastic Net Regularization Analysis

**DOI:** 10.1002/cnr2.70656

**Published:** 2026-09-30

**Authors:** Anna Giczewska, Krzysztof Pastuszak, Leon van Hout, Edward Post, Jip Ramaker, Ken Zwaan, Anna Supernat, Bart A. Westerman

**Affiliations:** ^1^ Laboratory of Translational Oncology, Intercollegiate Faculty of Biotechnology University of Gdańsk and Medical University of Gdańsk Gdańsk Poland; ^2^ Center of Biostatistics and Bioinformatics Medical University of Gdańsk Gdańsk Poland; ^3^ Department of Algorithms and System Modeling Gdansk University of Technology Gdańsk Poland; ^4^ Department of Neurosurgery, Cancer Center Amsterdam Amsterdam UMC Location VUMC Amsterdam the Netherlands; ^5^ Department of Human Genetics, Cancer Center Amsterdam Amsterdam UMC Location VUMC Amsterdam the Netherlands

**Keywords:** bioinformatics, biomarkers, glioblastoma, pathway analysis, platelet RNA

## Abstract

**Background:**

Glioblastoma (GBM) is the most aggressive form of primary brain cancer. Blood platelets have emerged as biomarkers where their RNA expression pattern indicates a tumor's presence. Our study aimed to characterize these patterns to identify biological processes associated with GBM and to evaluate their potential as a non‐invasive biomarker.

**Methods:**

We analyzed platelet RNA‐seq expression profiles from 84 GBM patients and 319 healthy individuals using two complementary analytical approaches of Gene Set Enrichment Analysis and Threshold‐based Differential Expression Analysis with Elastic Net regularization. Together, these approaches may offer complementary perspectives on biological pathways associated with GBM and the descriptive functional annotation of a predictive platelet RNA gene signature, providing insight into platelet‐tumor interactions.

**Results:**

Our GSEA broad analysis highlighted platelet‐related biological processes, including coagulation, wound healing, immune regulation, and cytoskeletal organization. The Elastic Net framework additionally suggested pathways related to immune activation, cell signaling, and protein folding. Overall, these findings may suggest that platelet‐derived RNA not only has potential as a non‐invasive biomarker but may also provide insight into the systemic biological processes associated with the GBM tumor microenvironment.

**Conclusions:**

Our data suggests that platelet‐derived RNA may reflect biological processes associated with the GBM tumor microenvironment, where wound repair and immune‐related processes may play important roles. Our findings provide a resource for further research into the mechanisms that may underlie the pathophysiology and progression of glioblastoma.

**Abbreviations**
AUC: area under the curve
B2M: beta‐2‐microglobulin
CAMK2D: calcium/calmodulin dependent protein kinase II delta
CBX7: Chromobox 7
CTCs: circulating tumor cells
DAMPs: damage‐associated molecular patterns
DESeq2: differential expression sequencing 2
EDTA: ethylenediaminetetraacetic acid
FDR: false discovery rate
GBM: glioblastoma
GO: Gene Ontology
GOMCL: Gene Ontology Markov Clustering
GSEA: gene set enrichment analysis
IDH: isocitrate dehydrogenase
LGALS2: Galectin 2
NF1: Neurofibromin 1
ORA: overrepresentation analysis
PAMPs: pathogen‐associated molecular patterns
PIK3IP1: phosphoinositide‐3‐kinase interacting protein 1
RNA‐seq: RNA sequencing
SMART‐seq: switching mechanism at 5′ end of RNA template for sequencing
TDEA: threshold‐based differential expression analysis
TLRs: Toll‐like receptors
TME: tumor microenvironment
VEGF: vascular endothelial growth factor

## 1 Introduction

Glioblastoma (GBM) is the most aggressive and common form of primary brain cancer, characterized by rapid tumor growth, diffuse infiltration, and poor prognosis, with a median survival time of approximately 15 months following diagnosis [1]. The complex etiology of GBM involves a combination of genetic mutations, epigenetic alterations, and dysregulated signaling pathways that drive unchecked tumor proliferation, diffuse invasion, and resistance to conventional therapies [2]. Notably, GBM is highly heterogeneous and typically has a relatively low tumor mutational burden, so individual mutations are rarely specific or deterministic. These detrimental factors are thought to be compounded by the tumor's microenvironment (TME), which plays a crucial role in modulating the immune response and promoting tumor progression [3]. This microenvironment, composed of immune cells, stromal components, and signaling molecules, is also influenced by platelets, which are increasingly recognized for their biological role in the TME [3, 4].

Platelets, traditionally known for their role in hemostasis, are increasingly understood to influence cancer progression, including GBM. They release pro‐angiogenic factors such as vascular endothelial growth factor (VEGF), contribute to the immune evasion of circulating tumor cells (CTCs), and interact with various components of the TME to promote tumor survival and spread [5]. These activities suggest that platelet‐derived RNA signatures may serve as valuable biomarkers for GBM, offering insights into the systemic effects of the tumor and potential therapeutic targets [6].

While much of the research on platelet‐derived RNA has focused on its utility in machine learning classifiers for cancer detection and monitoring [7], our study aims to delve deeper into the molecular mechanisms underlying GBM. By analyzing platelet‐derived RNA from GBM patients, we seek to identify dysregulated pathways that may reveal novel biological insights which could lead to therapeutic opportunities. To obtain a comprehensive and high‐level perspective on differential gene expressions, we employed two complementary analytical approaches, each offering a distinct focus: Gene Set Enrichment Analysis (GSEA) [8] and Threshold‐based Differential Expression Analysis (TDEA) [9] with Elastic Net Regularization [10]. In brief, Elastic Net Regularization fits a penalized regression model that simultaneously performs classification and feature selection by shrinking many coefficients toward zero while retaining a sparse set of most informative genes. Each method was subsequently followed by Gene Ontology (GO)‐based Over Representation Analysis (ORA) to further explore platelet‐derived RNA expression profiles in GBM patients compared to healthy individuals. Since performing ORA on the original gene set without any preselection of genes may lead to ambiguous findings, applying these selection methods first allowed us to refine the analysis and enhance the detection of meaningful pathway enrichments. GSEA and TDEA represent complementary analytical strategies that provide different perspectives on transcriptomic data [11]. GSEA evaluates coordinated changes across the full ranked gene list, enabling pathway‐level interpretation while accounting for multiple testing. In contrast, TDEA followed by Elastic Net regularization was used to derive a predictive platelet RNA signature by selecting genes that best discriminate GBM from healthy controls. Because genes selected by penalized regression primarily reflect predictive performance rather than direct mechanistic involvement [12], GO‐based ORA of the resulting 103‐gene signature was included only as descriptive functional annotation.

This complementary framework illustrates how pathway‐level analyses and predictive feature‐selection approaches can provide different but informative perspectives on the same platelet RNA‐seq dataset. By integrating these complementary analyses, our study provides additional insight into platelet‐derived RNA alterations associated with GBM and supports further investigation of platelet‐derived RNA as both a source of biological information and a potential non‐invasive biomarker for GBM, generating hypotheses for future diagnostic and therapeutic research.

## 2 Materials and Methods

### 2.1 Sample Selection

Our study re‐analyzes publicly available data from [13] (GEO accession number: GSE156902). Detailed information on sample collection and diagnostics can be found in the original study [13]. No new samples were collected for the present study and therefore did not require additional ethical approval [13].

Within the cohort of asymptomatic controls, individuals had no cancer diagnosis at the time of, or before, blood sample collection. However, it is important to note that no subsequent diagnostic tests were performed to verify the absence of cancer.

According to the WHO 2021 classification of CNS tumors [14], glioblastoma refers to IDH‐wildtype grade‐4 diffuse astrocytic tumors. Therefore, to ensure a molecularly consistent GBM cohort, patients with known astrocytoma grade IV were excluded from our analysis. Furthermore, we observed a strong batch effect during data pre‐processing for the healthy controls from the Netherlands Cancer Institute (as shown in Figure S1), likely caused by a technical issue during sample isolation, as discussed in [15]. As this subset was small, we chose to exclude these samples from downstream analysis rather than perform batch‐effect correction. In conclusion no batch‐effect correction was applied to the final dataset.

Therefore, from the original cohort of 89 GBM patients [13], 84 individuals diagnosed with GBM were selected in our study, along with 319 healthy controls. This resulted in the final study cohort of 403 donors (GBM and healthy controls) including 4412 genes. Where the 4412 genes represent the expressed‐gene set after low‐count filtering of the raw count matrix (genes retained if they had ≥ 30 raw counts in ≥ 10% of samples in at least one group), consistent with the gene‐filtering strategy previously reported for this dataset [16, 17]. All GBM patients included in this study were treatment‐naive at the time of blood collection.

### 2.2 Study Design

Because the underlying platelet RNA‐seq dataset has been published previously [13], the goal of the present study is to extend interpretation at the pathway level and evaluate how different analysis strategies influence biological conclusions. Summary of the added value was summarized in the Table S1.

Our study aimed to investigate platelet‐derived RNA alterations associated with GBM using two complementary analytical strategies designed to address different objectives. GSEA was applied to the complete ranked gene‐expression dataset to identify biological pathways associated with GBM at the transcriptome level. In parallel, TDEA followed by Elastic Net regularization was used to derive and evaluate a predictive platelet RNA signature capable of discriminating GBM patients from healthy controls. GO‐based ORA was subsequently applied to summarize the biological context of the results obtained from each approach, providing pathway‐level interpretation for the GSEA analysis and descriptive functional annotation for the Elastic Net‐derived gene signature.

Because these approaches address different analytical questions, they were considered complementary rather than directly comparable. Finally, publicly available transcriptomic datasets were analyzed to evaluate whether the principal biological programs identified in our platelet‐derived RNA analyses were consistently observed in independent datasets.

### 2.3 Data Processing and Analysis

In pursuit of gaining insight from our primary research method, we utilized GSEA to perform a comprehensive analysis of GBM and healthy cohort, aiming to identify a ranked subset of differentially expressed gene sets. GSEA enabled the prioritization of gene sets based on their expression patterns, highlighting those most relevant to our study. This approach was critical in ranking the entire pool of genes, without excluding any identified using the DESeq2 package. Gene Ontology categories were examined directly within the GSEA method to identify significance of the observed gene expression patterns. Given that GSEA results can depend on the random seed, particularly with large input gene sets, we performed 10 independent iterations with different random seeds to assess the consistency of the enrichment results. To reduce redundancy among enriched GO terms, GO‐term clustering was subsequently performed on the enriched GO terms from a single GSEA analysis using the GOMCL framework [18], which groups GO terms based on shared annotated genes. For visualization purposes, representative GO cluster labels were selected using a combined score reflecting enrichment magnitude and statistical significance. As an exploratory descriptive analysis, we also performed TDEA using DESeq2 to summarize differentially expressed platelet RNA transcripts between GBM patients and healthy controls. Genes were selected using an FDR‐corrected *p*‐value < 0.05, resulting in 2126 genes. These results are provided in Figure S2 for reference. This exploratory analysis was included to provide further insight but was not used for TDEA with Elastic Net workflow and was not subject to further interpretation in the manuscript.

For our second method, the TDEA with Elastic Net workflow, we adhered to a stringent 60/40 split, ensuring a well‐controlled and balanced division of the study cohort (training set: 60%; 51 GBM and 192 healthy individuals, test set: 40%; 33 GBM and 127 healthy individuals). Subsequently, TDEA was performed utilizing DESeq2 on 4412 genes within the training set to determine differentially expressed genes between GBM and healthy donors. Following the application of a threshold of 1.3‐fold change and a 0.1 FDR‐corrected *p*‐value, a baseline set of 323 differentially expressed genes was identified. These 323 candidate genes were then used as predictors in an Elastic Net‐penalized logistic regression model, with disease status, GBM versus healthy control, as the binary outcome. Model tuning was performed using five‐fold cross‐validation repeated five times within the training set. In the first Elastic Net model, all 323 TDEA‐selected candidate genes were included. Genes were then ranked according to the absolute value of their Elastic Net coefficient. Genes with an absolute coefficient greater than 0.05 were retained, resulting in the final 103‐gene set. Subsequently, a second Elastic Net model was trained using this reduced 103‐gene set. The independent test set was kept separate throughout model development and was used only for the final evaluation of model performance. Model performance was evaluated on the test set using predicted probabilities to calculate the AUC. A confusion matrix was also generated to summarize classification performance in the test set. Subsequently, GO‐based ORA was conducted using the final 103‐gene set derived from the TDEA with Elastic Net workflow as descriptive functional annotation of the predictive platelet RNA signature.

To further validate the consistency of our findings, GSEA was performed on publicly available external bulk and single‐cell datasets using pseudobulk analysis to investigate the relationships between enriched pathways in the GO and Reactome databases, as well as across different tumor grades and sequencing methods (10× and SMARTseq). Enrichment scores, Pearson correlation coefficients, percentages of variance explained, and *p*‐values were calculated to evaluate the alignment and relationships of pathway enrichment between the two annotation systems.

## 3 Results

### 3.1 Using GSEA to Illuminate Complex Molecular Pathways in GBM Disease Mechanisms

In our research study, we aimed to investigate the molecular mechanisms underlying platelet‐tumor interactions in GBM by analyzing platelet‐derived RNA expression patterns. Figure 1A illustrates our study design, where platelet samples from 84 GBM patients and 319 healthy controls were analyzed. Figure 1B outlines the sequential steps followed in the study design for analyzing platelet RNA‐Seq profiles in GBM patients and healthy controls. Each of the three sections represents the stage of the analysis, respectively the source data, the feature selection stage where GSEA or the TDEA and Elastic Net Regularization, and the GO‐based ORA analysis were applied, where each selected gene subset was analyzed separately to identify enriched pathways. This layout reflects the study's progression from data acquisition to feature selection and pathway analysis, enhancing the clarity of the methodological flow.

**FIGURE 1:**
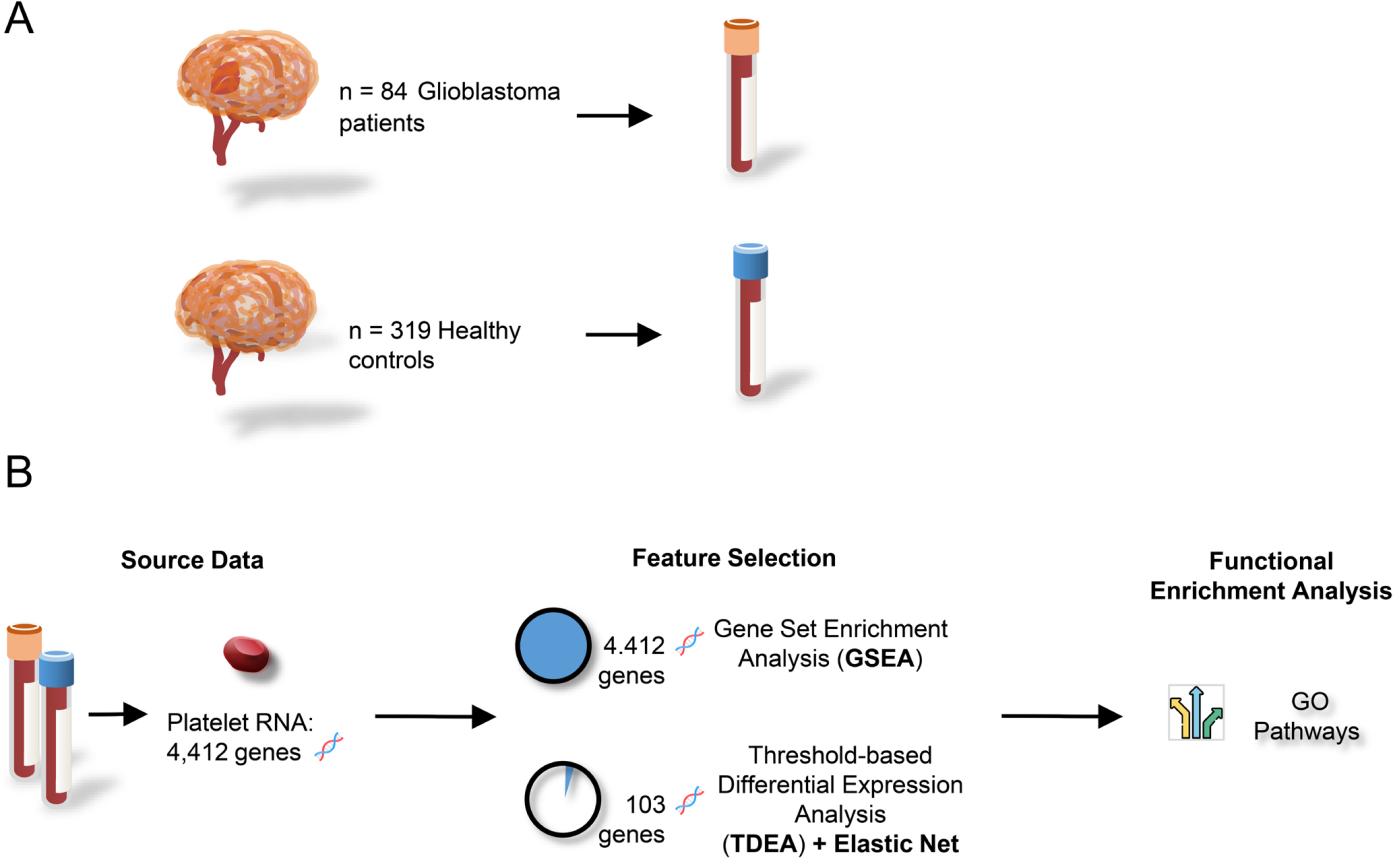
(A) Study design, including the analysis of platelet RNA expression patterns from 84 GBM patients and 319 healthy controls. (B) Sequential workflow of the study, detailing the stages of analysis: Data acquisition, feature selection using GSEA, TDEA, Elastic Net Regularization, and GO pathway analysis. Each selected gene subset was analyzed independently to identify enriched pathways, reflecting the progression from data collection to feature selection and pathway enrichment. This comprehensive layout captures the methodological flow of the study.

In our first approach, GSEA was applied to all expressed genes (Table S2), generating a ranked gene list that captured platelet RNA expression patterns associated with GBM. GO pathways were then evaluated directly within the GSEA framework, as outlined in Figure 2A. The resulting enrichment analysis is summarized in the dot plot shown in Figure 2B. The dot plot revealed numerous significantly enriched pathways associated with GBM. Positively enriched pathways (shown in red) included platelet‐related processes such as blood coagulation, platelet activation, wound healing, and vascular and circulatory functions. Additional enriched terms reflected cytoskeletal organization, cell adhesion, extracellular matrix organization, and actin‐related processes (shown in blue).

**FIGURE 2:**
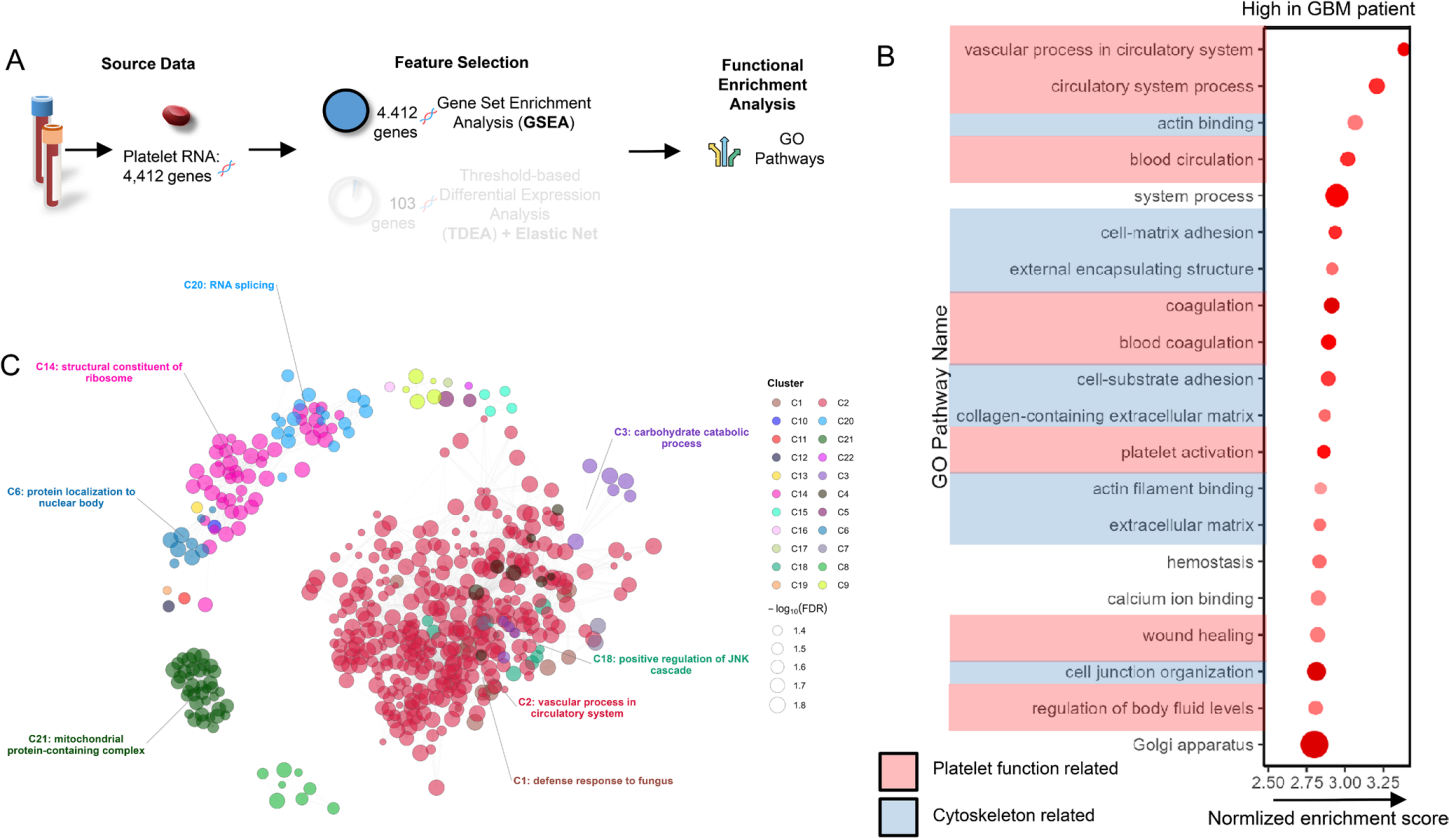
(A) Overview of the study workflow. Platelet RNA‐Seq data from 4412 genes were subjected to feature selection using GSEA. These genes were further analyzed through GO‐based ORA to identify enriched pathways. (B) GO pathway analysis showing the top enriched pathways positively associated with GBM patients. Pathways are ranked based on their normalized enrichment score, with red indicating platelet function‐related pathways and blue indicating cytoskeleton‐related pathways. (C) GO‐term similarity network generated using the GOMCL framework. Nodes represent enriched GO terms grouped according to shared annotated genes and colored by cluster membership. Representative GO labels for eight largest clusters were selected using a combined score reflecting enrichment magnitude and statistical significance are shown for visualization. Complete cluster membership is provided in Table 1 and Table S5.

To facilitate interpretation of the enriched GO terms, GO‐term clustering was performed on the enriched pathways from a representative GSEA analysis using the GOMCL framework, which groups GO terms according to overlap in their annotated genes (Figure 2C). This analysis identified 22 GO‐term clusters, reducing redundancy among related GO annotations. For visualization purposes, each cluster was labeled using the GO term with the highest representative score (|NES| × −log_10_(FDR‐adjusted *p*‐value)). Because individual clusters frequently encompassed multiple related biological processes, this score was used only to assign label to representative 8 largest clusters to highlight prominent biological themes rather than fully describe clusters. Additional cluster descriptors, including the largest GO term, the GO term with the lowest FDR‐adjusted *p*‐value, the most connected GO term, and the representative GO term used for labeling, are summarized in Table 1, while the complete cluster composition is provided in Table S5. Additionally, a GO‐term similarity matrix illustrating relationships among clustered GO terms is presented in Figure S3.

**TABLE 1:** Summary of GO‐term clusters generated using the GOMCL framework.

Cluster	# of GOs	# of genes	Largest GO term (*x*/*n*, *p*)	GO term with smallest *p* (*x*/*n*, *p*)	Most connected GO term (*x*/*n*, *p*)	GO term with highest rep_score (*x*/*n*, rep_score)	NES direction (mostly)
**C1**	**15**	**257**	**Secretory vesicle (120/427, 1.45e−02)**	**Secretory vesicle (120/427, 1.45e−02)**	**Secretory granule (113/374, 1.45e−02)**	**Defense response to fungus (9/14, 4.71)**	**Positive**
**C2**	**315**	**1778**	**Regulation of biological quality (435/796, 1.45e−02)**	**Regulation of biological quality (435/796, 1.45e−02)**	**Regulation of biological quality (435/796, 1.45e−02)**	**Vascular process in circulatory system (38/58, 6.20)**	**Positive**
**C3**	**10**	**151**	**Carbohydrate metabolic process (106/162, 1.55e−02)**	**Carbohydrate catabolic process (44/56, 1.45e−02)**	**Carbohydrate metabolic process (106/162, 1.55e−02)**	**Carbohydrate catabolic process (44/56, 4.68)**	**Positive**
C4	8	218	Lipid metabolic process (192/339, 1.55e−02)	Lipid metabolic process (192/339, 1.55e−02)	Lipid metabolic process (192/339, 1.55e−02)	Lipid biosynthetic process (141/194, 3.98)	Positive
C5	2	9	T cell receptor complex (9/10, 2.65e−02)	Alpha‐beta T cell receptor complex (4/5, 1.62e−02)	Alpha‐beta T cell receptor complex (4/5, 1.62e−02)	Alpha‐beta T cell receptor complex (4/5, 4.60)	Negative
**C6**	**9**	**114**	**RNA localization (59/112, 4.80e−02)**	**Protein localization to nuclear body (10/11, 1.65e−02)**	**Protein localization to nuclear body (10/11, 1.65e−02)**	**Protein localization to nuclear body (10/11, 4.47)**	**Negative**
C7	3	189	Autophagy (161/254, 1.65e−02)	Autophagy (161/254, 1.65e−02)	Autophagy (161/254, 1.65e−02)	Autophagy (161/254, 3.83)	Positive
C8	8	30	B cell proliferation (21/30, 1.84e‐02)	Gamma‐delta T cell activation (8/10, 1.65e−02)	Negative T cell selection (4/5, 2.84e−02)	B cell proliferation (21/30, 5.00)	Negative
C9	5	12	MHC class II protein complex binding (11/18, 4.95e−02)	CD4‐positive, CD25‐positive, alpha‐beta regulatory T cell differentiation (2/3, 1.96e−02)	CD4‐positive, CD25‐positive, alpha‐beta regulatory T cell differentiation (2/3, 1.96e−02)	CD4‐positive, CD25‐positive, alpha‐beta regulatory T cell differentiation (2/3, 3.60)	Negative
C10	1	21	Cajal body (21/25, 2.48e−02)	Cajal body (21/25, 2.48e−02)	Cajal body (21/25, 2.48e−02)	Cajal body (21/25, 4.07)	Negative
C11	1	10	Chemokine receptor binding (10/11, 3.12e−02)	Chemokine receptor binding (10/11, 3.12e−02)	Chemokine receptor binding (10/11, 3.12e−02)	Chemokine receptor binding (10/11, 3.42)	Negative
C12	1	21	Mitochondrial intermembrane space (21/27, 3.18e−02)	Mitochondrial intermembrane space (21/27, 3.18e−02)	Mitochondrial intermembrane space (21/27, 3.18e−02)	Mitochondrial intermembrane space (21/27, 3.40)	Negative
C13	1	77	Catalytic activity, acting on RNA (77/116, 3.33e−02)	Catalytic activity, acting on RNA (77/116, 3.33e−02)	Catalytic activity, acting on RNA (77/116, 3.33e−02)	Catalytic activity, acting on RNA (77/116, 3.41)	Negative
**C14**	**50**	**309**	**ncRNA metabolic process (104/190, 4.88e−02)**	**Large ribosomal subunit rRNA binding (2/3, 1.57e−02)**	**Cytoplasmic translation (100/120, 3.34e−02)**	**Structural constituent of ribosome (69/82, 13.15)**	**Negative**
C15	4	4	Positive regulation of pyroptosis (3/4, 3.77e−02)	AIM2 inflammasome complex assembly (2/3, 3.70e−02)	Cytolysis (2/3, 3.70e−02)	Positive regulation of pyroptosis (3/4, 2.96)	Negative
C16	1	64	Ribonucleoprotein complex binding (64/76, 3.85e−02)	Ribonucleoprotein complex binding (64/76, 3.85e−02)	Ribonucleoprotein complex binding (64/76, 3.85e−02)	Ribonucleoprotein complex binding (64/76, 3.31)	Negative
C17	1	23	Polyubiquitin modification‐dependent protein binding (23/31, 4.27e−02)	Polyubiquitin modification‐dependent protein binding (23/31, 4.27e−02)	Polyubiquitin modification‐dependent protein binding (23/31, 4.27e−02)	Polyubiquitin modification‐dependent protein binding (23/31, 2.95)	Positive
**C18**	**9**	**596**	**Catalytic activity, acting on a protein (465/681, 1.45e−02)**	**Catalytic activity, acting on a protein (465/681, 1.45e−02)**	**Protein serine/threonine kinase activity (99/145, 4.31e−02)**	**Positive regulation of JNK cascade (19/23, 3.93)**	**Positive**
C19	1	6	AMP biosynthetic process (6/7, 4.41e−02)	AMP biosynthetic process (6/7, 4.41e−02)	AMP biosynthetic process (6/7, 4.41e−02)	AMP biosynthetic process (6/7, 2.97)	Negative
**C20**	**19**	**256**	**RNA splicing, via transesterification reactions (132/162, 4.44e−02)**	**Spliceosomal complex assembly (38/46, 2.05e−02)**	**RNA splicing, via transesterification reactions (132/162, 4.44e−02)**	**RNA splicing, via transesterification reactions (132/162, 6.27)**	**Negative**
**C21**	**40**	**174**	**Organelle inner membrane (97/169, 4.58e−02)**	**Proton‐transporting ATP synthase complex, coupling factor F(o) (9/10, 1.65e−02)**	**Organelle inner membrane (97/169, 4.58e−02)**	**Mitochondrial protein‐containing complex (80/110, 7.37)**	**Negative**
C22	1	158	Chromosomal region (158/171, 4.59e−02)	Chromosomal region (158/171, 4.59e−02)	Chromosomal region (158/171, 4.59e−02)	Chromosomal region (158/171, 3.34)	Negative

*Note:* Cluster descriptors include the number of GO terms and annotated genes, the largest GO term, the GO term with the lowest FDR‐adjusted *p*‐value, the most connected GO term, and the representative GO term. Bold indicates the eight largest GO‐term clusters, based on the number of GO terms assigned to each cluster, selected for labeling in Figure 2c.

Furthermore, to evaluate the robustness of the GSEA results, the enrichment analysis was repeated using 10 different random seeds. The resulting pathways showed a high degree of consistency across iterations, supporting the stability of the identified biological processes (results summarized in Tables S3 and S4). GO‐term clustering was performed only for the single GSEA analysis described above and was not repeated for results from each iteration.

### 3.2 Unraveling Gene Expression and Pathways Disparities Between GBM and Healthy Individuals Through TDEA and Elastic Net Regularization Analysis

In the examination of our full study cohort, an initial computational analysis was executed through the DESeq2 algorithm. This analytical approach aimed to delineate the subset of genes exhibiting the most significant differential expression in the comparison between GBM and healthy individuals. Through applying TDEA with a standard threshold (FDR < 0.1, fold change > 1.3, and median log_2_ expression > 3) we identified a specific set of most important genes. For a comprehensive exploration of the details associated with gene expression, the exhaustive list and additional relevant information are accessible in (Figure S2), providing a thorough resource for further investigation and interpretation.

In the next step our dataset was divided into training (50 GBM, 191 healthy) and test sets, with TDEA performed within the training set identifying 323 candidate genes differentiating GBM patients from healthy controls. These genes were used as input for an Elastic Net‐penalized logistic regression model trained to classify GBM versus healthy‐control samples. After model tuning by repeated five‐fold cross‐validation, genes were ranked by the absolute value of their Elastic Net coefficient. Applying a coefficient threshold of > 0.05 resulted in a final set of 103 genes. The final Elastic Net model based on this 103‐gene set was then evaluated in the independent test set using predicted probabilities. The model achieved an AUC of 0.99 and a 5% misclassification rate (Table S6 and Figure S4). To further assess robustness of the AUC estimate, we repeated the procedure across 10 random seeds (mean AUC = 0.989, SD = 0.006, mean Sensitivity = 0.877, SD = 0.068, mean Specificity = 0.980, SD = 0.008). To further place this performance into context, we compared the selected 103‐gene signature with 100 randomly generated 103‐gene signatures evaluated using the same modeling and independent test‐set framework. Although randomly selected gene sets also demonstrated substantial predictive performance, the original 103‐gene signature consistently outperformed the random reference distribution in both test AUC and balanced accuracy (Figure S5). Additionally, we evaluated the 103‐gene signature in an independent publicly available platelet RNA‐seq cohort [7]. No genome‐wide feature‐selection step was performed in the external cohort. This external assessment showed lower but still strong discriminatory performance (AUC = 0.93), supporting the potential transferability of the selected platelet RNA signature. The GBM and healthy study cohorts, encompassing both the training and test sets, featuring a curated set of 103 most important genes, were subjected to GO enrichment analysis. No GO terms remained statistically significant after multiple‐testing correction. Therefore, the top GO terms shown in Figure 3 are ranked by nominal *p*‐value and are presented only as descriptive functional annotation of the predictive gene signature. This analysis yielded the identification of 1778 signaling pathways with our exclusive emphasis only on the top 20 pathways (see Figure 3). The resulting pathways manifest notable diversity and reflect various cellular and molecular processes. These processes include responses to bacterial infection and external stimuli, cell signaling and migration, immune cell activation, gene regulation, and protein folding, highlighting the importance of both immune‐related functions and cellular maintenance activities.

**FIGURE 3:**
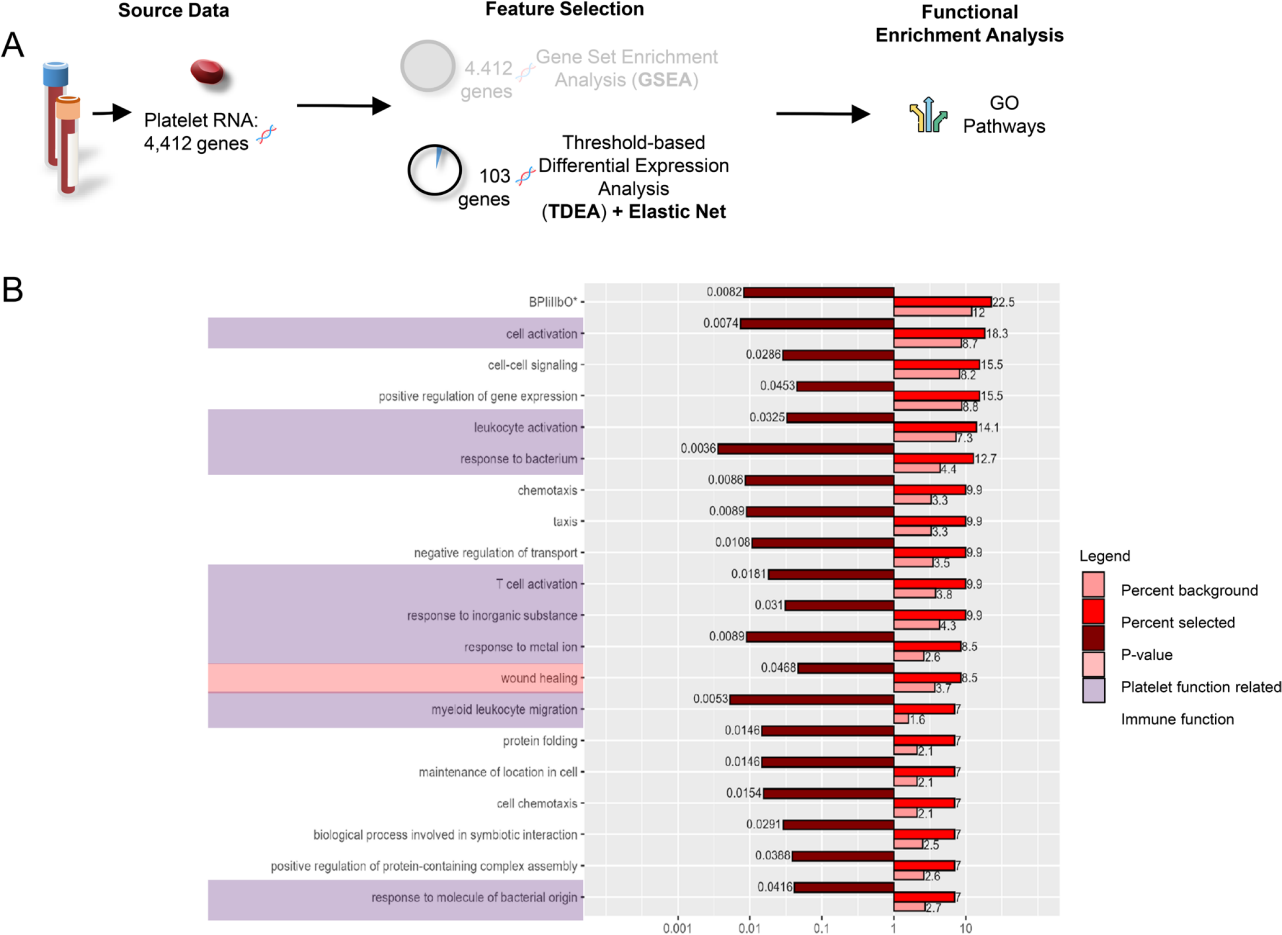
(A) Overview of the study workflow. Platelet RNA‐Seq data from 103 genes were subjected to feature selection using TDEA followed by Elastic Net Regularization. These genes were further analyzed through GO‐based ORA for descriptive annotation. (B) Top 20 nominally ranked GO terms identified by GO‐based ORA of the Elastic Net‐derived 103‐gene predictive signature. The percentage background refers to the proportion of all genes within a given dataset that are associated with a particular pathway or biological process. This background typically represents the entire set of genes under study. The percentage selected represents the proportion of genes within the selected subset of genes that are specifically identified as differentially expressed or being involved in the process of interest. These GO pathways are presented as descriptive functional annotation. Pathways highlighted in red are associated with platelet function, while those in purple are related to immune function (*BPIiIIbO = biological process involved in interspecies interaction between organisms).

In Figure 3 alongside the depicted *p*‐values denoted in dark red, we have delineated the percentage background (in light red) and the percentage selected (in red). When comparing the percentage selected to the percentage background, significantly elevated values of the percentage selected relative to the percentage background suggest that a higher‐than‐expected number of genes associated with the pathway of interest are present in the dataset. This discrepancy indicates potential enrichment of the pathway within the dataset, implying that it may play a significant role in differentiating GBM and healthy individuals. Because these GO terms are based on nominal *p*‐values, they are presented only as descriptive functional annotation and should be interpreted with caution.

### 3.3 Bulk and Semi‐Bulk Analysis Confirm Ontologies Identified Through Platelet‐Derived RNA Profiling

To assess whether the strongest platelet‐related biological signals identified in our primary platelet‐derived RNA analyses are also supported by independent datasets, we performed an external consistency analysis using publicly available bulk and single‐cell tumor transcriptomic data. Because independent platelet‐derived RNA cohorts with comparable preprocessing and sufficient clinical annotation are limited in the public domain, we focused on validating the biological programs most consistently implicated by our platelet analyses, namely wound healing and platelet activation. We prioritized wound healing and platelet activation because these represent core platelet functions and were among the most consistently enriched platelet‐related processes across both our GSEA‐based overview and the more stringent TDEA/Elastic Net workflow. We used glioma datasets because high‐quality external tumor transcriptomic datasets with sufficient sample size and appropriate annotation were available primarily under the broader “glioma” label rather than exclusively GBM. This analysis is intended as supportive evidence for the biological coherence of key pathways highlighted by platelet‐derived RNA profiling, rather than a direct external replication of the full platelet‐derived RNA differential expression model.

To build upon our previous findings, we analyzed bulk RNA expression data (GEO accession number: GSE16011) from glioma patients across various grades, obtained from [19]. Using this data, we generated specific signatures and identified a strong correlation between platelet activation/aggregation and wound healing signatures (Figure 4A). Furthermore, the analysis of gliomas by grade revealed that wound healing signatures are elevated in glioma patients compared to normal controls (Figure 4B). While our study primarily focuses on GBM, we have also provided results for other glioma grades for reference.

**FIGURE 4:**
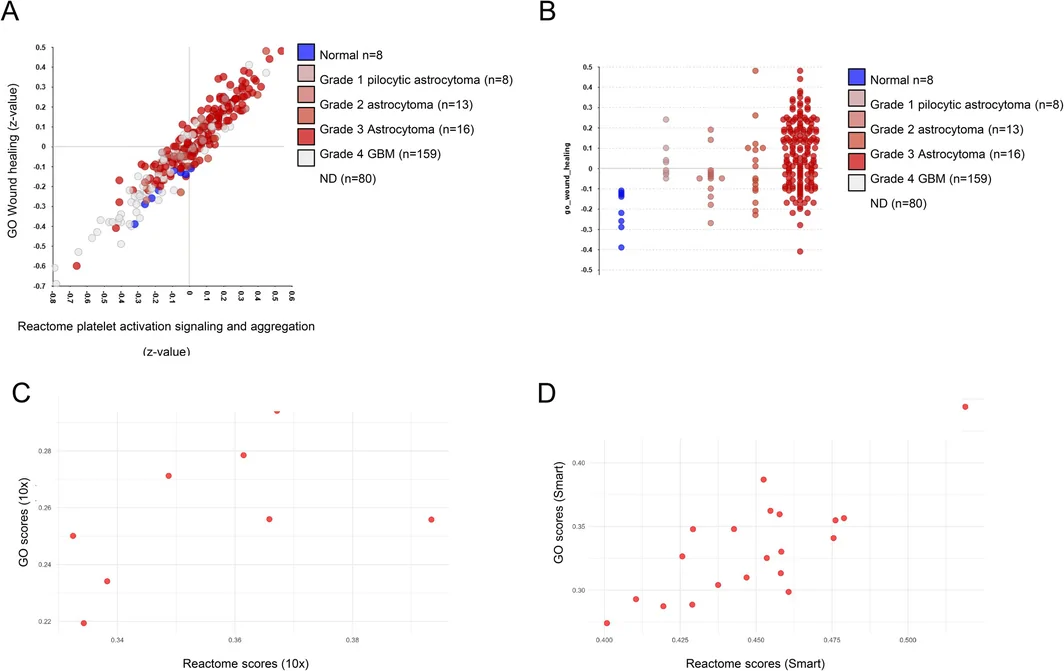
(A) Correlation between platelet activation/aggregation and wound healing signatures in bulk RNA expression data from glioma patients of varying grades. (B) Comparison of wound healing signature levels across glioma grades and normal controls, showing elevated expression in glioma patients. (C) Scatterplot illustrating the relationship between GO wound healing scores (*y*‐axis) and Reactome platelet activation, signaling, and aggregation scores (*x*‐axis) across nine samples analyzed using 10× single‐cell data. Each point represents an individual sample. (D) Scatterplot illustrating the relationship between GO wound healing scores (*y*‐axis) and Reactome platelet activation, signaling, and aggregation scores (*x*‐axis) across 20 samples analyzed using SMART‐Seq data. Each point represents an individual sample.

Furthermore, a semi‐bulk approach was taken to analyze single‐cell RNA sequencing data (GEO accession number: GSE131928) from [20], we used only samples collected from adults. In this analysis, we observed a positive correlation between platelet activation/aggregation and wound healing signatures across samples analyzed using the 10× Genomics platform (Figure 4C). Similarly, analysis of single‐cell data generated using the SMART‐Seq platform revealed an even stronger correlation between these pathways (Figure 4D). These correlative findings further support the potential interplay between platelet activation and wound healing in the glioma tumor microenvironment, highlighting the robustness of this relationship across different sequencing methods.

## 4 Discussion

We analyzed RNA‐seq data from blood platelets of GBM patients and compared them to healthy control samples to investigate systemic effects of GBM on gene expression. By employing GO‐based ORA, GSEA, and TDEA with Elastic Net regularization, we identified dysregulated pathways reflective of both consistent and divergent biological processes associated with GBM. These data show that blood‐based biomarkers, in particular platelet RNA, can provide insight into GBM pathophysiology and developing non‐invasive diagnostic strategies.

Through GSEA, we identified pathways normally associated with platelet functions, such as blood coagulation, wound healing, and cytoskeletal organization. GO‐term clustering further organized the enriched pathways into broader biological themes including vascular processes, RNA processing, ribosome‐associated functions, immune regulation, and cellular metabolism. Pathways such as VEGF signaling and platelet aggregation were also enriched. These findings suggest that platelets, traditionally associated with blood clotting, may also play an active role in modifying the tumor microenvironment through systemic reprogramming [21] and enhanced angiogenesis and tumor‐platelet interactions [22]. Together, these results suggest that GBM may be associated with alterations in platelet functionality linked to tumor‐promoting processes. While biologically plausible, these pathway‐level associations do not establish causality or GBM‐specific mechanisms.

Elastic Net‐based TDEA identified predictive gene signature, for which descriptive GO annotation suggested categories related to immune activation, responses to external stimuli, cell signaling, and protein folding. Although these GO terms should be interpreted with appropriate caution and viewed as descriptive functional annotation, they may provide functional context for the predictive gene signature and are consistent with biological processes previously implicated in immune evasion mechanisms [23]. However, potential links to immune modulation remain speculative without matched tumor tissue or functional assays. Although indirect, the recruitment of neutrophils and monocytes might be linked to leukocyte activation and chemotaxis, possibly shaping a pro‐inflammatory environment to support tumor growth. Although none of these mechanistic associations are directly proven, these findings align with previous evidence suggesting that platelets are key modulators of the immune landscape, not only facilitating immune suppression but also promoting inflammation that supports GBM progression [21, 22, 23, 24].

Pathways related to bacterial responses were particularly intriguing, as none of the GBM patients in our study exhibited signs of infection at the time of sample collection. This observation led us to explore alternative mechanisms, such as the involvement of Damage‐Associated Molecular Patterns (DAMPs) [25]. DAMPs, which are released from necrotic cells, are known to activate platelet Toll‐like receptors (TLRs), thereby mimicking bacterial responses and promoting systemic inflammation [26]. This mechanism might reflect the complex interplay between tissue damage, immune activation, and platelet‐mediated responses in GBM. Notably, DAMPs mimic Pathogen‐Associated Molecular Patterns (PAMPs), triggering immune responses that include platelet activation [27]. This may contribute to bacterial infection‐like signals in GBM tumors. DAMP signals may contribute to the inflammatory environment, promoting immune evasion and further suggesting a possible dual role of platelets in driving tumor progression and immune dysfunction.

Between GSEA and TDEA followed by Elastic Net analyses, we observed differences in the biological information provided by each approach. The FDR‐corrected GSEA analysis identified pathway‐level alterations associated with platelet biology, particularly in angiogenesis, immune modulation, and cytoskeletal remodeling. Additionally, GSEA identified downregulation of transcripts associated with ribosomal function and protein synthesis, aligning with previous findings [28] that highlight suppression of ribosome‐associated pathways in GBM‐related platelet‐derived RNA signatures. In contrast, Elastic Net identified a predictive 103‐gene platelet RNA signature. Descriptive GO annotation of this signature suggested categories related to immune activation, responses to external stimuli, cell signaling, and protein folding, providing functional context for the predictive gene set. Together, these complementary analyses provide both pathway‐level biological interpretation and predictive insight into platelet‐derived RNA alterations associated with GBM.

The relationships among the GSEA‐identified pathways suggest how systemic processes—such as immune activation, inflammation, and angiogenesis—could potentially shape the GBM tumor microenvironment. Often described as a chronically regenerating wound, GBM relies on persistent immune activation and inflammation to sustain tumor progression [29]. This continuous immune infiltration, driven by myeloid cells such as macrophages and microglia, fuels angiogenesis, immune evasion, and chronic tissue remodeling. Additionally, reactive gliosis—a hallmark of GBM—further supports immune infiltration and inflammation, particularly when linked to NF1 loss, worsening tumor‐supportive processes and driving malignancy [30]. These findings show the complex dynamics between tumor and host cells that fuel GBM's aggressive behavior.

While these results provide valuable insights into the systemic changes associated with GBM, it is important to acknowledge that they are derived from platelet RNA rather than direct tumor samples. Consequently, these findings likely reflect systemic platelet responses associated with GBM rather than the intrinsic genetic complexities of the tumor itself.

In addition to the pathway‐level GSEA findings, our Elastic Net‐based analysis identified genes with potential value as a predictive non‐invasive platelet RNA signature for GBM. Immune‐related genes, such as *B2M* and *LGALS2*, highlight mechanisms of immune evasion [31, 32, 33], while downregulated genes such as *CXCR2P1* and *OAS1* suggest potential avenues for enhancing anti‐tumor immune responses [33, 34, 35]. Genes involved in cell proliferation (*CAMK2D*, *PIK3IP1*) [36, 37] and metabolism (*MAOB*, *FKBP5*) [38, 39, 40] further illustrate the multifaceted nature of GBM pathology. Dysregulated epigenetic regulators, such as *CBX7* and non‐coding RNAs, might underscore the importance of chromatin remodeling in tumor progression [41, 42, 43, 44, 45]. Collectively, these genes may provide useful predictive information; however, their direct mechanistic relevance to GBM cannot be inferred from Elastic Net selection alone and requires further functional validation. By categorizing these genes based on systemic processes, we establish a foundation for future preclinical studies aimed at developing targeted therapies and diagnostic tools based on platelet‐derived RNA signatures.

While our study provides valuable insights, we acknowledge its limitations. Specifically, sample selection bias, a modest number of GBM cases, and the arbitrary but commonly used cut‐offs for gene set selection may affect the generalizability of our results. Although our thresholds were chosen to balance sensitivity and the ability to detect relevant signals, we recognize that more conservative criteria could reduce the risk of false positives. In addition, incomplete treatment and clinical annotation limited our ability to assess potential confounding effects on platelet‐derived RNA signatures. Furthermore, GBM tumor heterogeneity could influence platelet‐derived RNA signatures and introduce additional variability, but our dataset was insufficiently annotated to evaluate this systematically. Although samples were collected prior to treatment initiation, residual confounding by other medications or clinical factors affecting platelet biology cannot be excluded. Therefore, these signatures should not be interpreted as strictly GBM‐specific without further confirmation in fully annotated cohorts. Moreover, the intricate interplay between GBM and platelet‐derived RNA signatures introduces additional complexity, as these associations are influenced by a range of tumor‐specific and microenvironmental factors that are not yet fully understood. Furthermore, because many platelet activation signatures are shared across cancer and inflammatory states, these findings should not be interpreted as GBM‐specific mechanisms, and functional inference about the tumor microenvironment should be considered hypothesis‐generating. Additionally, because the genes in the 103‐gene panel were selected using a penalized logistic regression framework, its genes are best interpreted as predictive features, while their possible mechanistic relevance to GBM should be considered hypothesis‐generating and require further validation. In addition, GO‐based ORA of the 103‐gene panel was used primarily as descriptive functional annotation of the predictive gene signature, and these pathway‐level findings should be interpreted as exploratory rather than as definitive evidence of pathway overrepresentation. A further limitation is that GO/pathway annotations are curated largely from studies in nucleated cells. Therefore, enrichment of nuclear or mitotic terms in platelet RNA should be interpreted with caution.

Despite the limitations of our study, our findings align with previous hypotheses suggesting that RNA signatures in TEPs could serve as a noninvasive tool for cancer detection and monitoring, supporting the potential of platelet‐derived RNA in predicting GBM [7, 46]. However, the precise mechanism remains uncertain and requires additional investigation in future studies.

## 5 Conclusions

This study demonstrates systemic alterations in platelet‐derived RNA associated with GBM, highlighting disease‐related changes in the circulating platelet transcriptome. Using complementary computational approaches, we identified pathway‐level biological processes associated with GBM and a platelet RNA gene signature with strong predictive performance. Together, these findings provide insight into systemic platelet responses associated with GBM and support the continued investigation of platelet‐derived RNA as a non‐invasive biomarker.

The identification of immune‐related pathways, particularly those involving leukocyte recruitment and chemotaxis, supports a potential role for platelets in systemic immune and inflammatory responses associated with GBM. Moreover, the involvement of DAMP‐mediated platelet activation suggests a possible mechanism by which GBM may promote systemic inflammation, although these observations require further validation.

While platelet‐derived RNA offers valuable insights into the systemic effects of GBM, further research is needed to validate these findings in larger cohorts and explore the link between blood‐based markers and localized tumor biology. Overall, our findings contribute to the growing understanding of platelet‐derived RNA alterations in GBM while generating hypotheses for future studies investigating platelet biology, disease mechanisms, and blood‐based diagnostic approaches.

## Author Contributions

**Edward Post:** data curation, resources. **Leon van Hout:** data curation, investigation, methodology, resources. **Jip Ramaker:** data curation, resources. **Bart A. Westerman:** conceptualization, methodology, investigation, supervision, funding acquisition, writing – review and editing, visualization. **Anna Giczewska:** conceptualization, formal analysis, investigation, methodology, software, writing – review and editing, writing – original draft, visualization, project administration, validation. **Krzysztof Pastuszak:** data curation, conceptualization, writing – review and editing, investigation. **Anna Supernat:** conceptualization, writing – review and editing, methodology, investigation, supervision, funding acquisition. **Ken Zwaan:** data curation, resources.

## Funding

This work was supported by the Health~Holland Grants called AI‐IMPACT (to B.A.W.) and The Toxicity Atlas (to B.A.W. and L.v.H.), and the Excellence Initiative “Research University” Grant (MNISW 07/IDUB/2019/94; to K.P.).

## Ethics Statement

The present analysis used publicly available anonymized data and therefore did not require additional ethical approval.

## Conflicts of Interest

A.S. and K.P. has one patent approved (P.435990) and filed two more patent applications related to platelet‐based diagnostics. The remaining authors declare no conflicts of interest.

## Supporting information


**Table S1:** Summary table comparing previously reported findings from the original platelet RNA‐seq study with the scope and contribution of the present re‐analysis.

**Table S2:** Comprehensive list of genes utilized in GSEA, including flagged genes used for TDEA in comparison between GBM and healthy controls. The table includes the mean fold change, log_2_ fold change, raw *p*‐values, and FDR‐corrected *p*‐values for each gene.

**Table S3:** Upregulated pathways summarized across 10 independent iterations using different random seeds. In each run, the top 20 positively enriched pathways were retained and assigned rank‐based weights from 2.0 to 0.1. Count over 10 iterations indicates the number of runs in which a pathway appeared among the top 20, and Averaged Rating represents the mean assigned weight across those occurrences.

**Table S4:** Downregulated pathways summarized across 10 independent iterations using different random seeds. In each run, the top 20 negatively enriched pathways were retained and assigned rank‐based weights from 2.0 to 0.1. Count over 10 iterations indicates the number of runs in which a pathway appeared among the top 20, and Averaged Rating represents the mean assigned weight across those occurrences.

**Table S5:** Summary of GO‐term clusters generated using the GOMCL framework. Cluster descriptors include the number of GO terms and annotated genes, the largest GO term, the GO term with the lowest FDR‐adjusted *p*‐value, the most connected GO term, the representative GO term and detailed list of pathways components in each cluster.

**Table S6:** Selected final gene set for TDEA followed by Elastic Net comparing GBM and healthy controls. The table includes model coefficients for each gene.

**Figure S1:** Boxplot analysis of gene expression in NKI and non‐NKI groups for various markers. The data show the distribution of expression levels for genes including PTPRC, CD3D, CD6, CD19, KLF1, and HB, with comparisons between the NKI and non‐NKI groups. Each plot represents the median, interquartile range, and outliers for each group.
**Figure S2:** (a) Overview of the study workflow. Platelet RNA‐Seq data from 2126 genes were subjected to feature selection using TDEA. These genes were further analyzed through GO‐based ORA to identify enriched pathways. (b) Significant pathways identified through GO‐based ORA of the entire population using the standard TDEA, ranked by significance with the most relevant pathways shown at the top. This analysis revealed enrichment (*q*‐value < 0.05) in 115 pathways, focusing on the top 20 pathways based on the gene‐to‐pathway ratio. In the figure, the *q*‐values (depicted in yellow) are shown alongside the background ratio (in blue) and the gene ratio (in green). The *q*‐value is a measure of statistical significance, with lower values meaning the result is more likely to be important and not due to random chance. Gene Ratio is based on significant genes identified using DESeq2. It shows how many of these significant genes are involved in each biological process. The Background Ratio, on the other hand, is based on all the genes considered during the analysis, which in our study includes all expressed genes. This ratio indicates how common those genes are in the overall considered gene pool. For a specific biological process, a Gene Ratio of 22.8 indicates that 22.8% of the significant genes identified are involved in this process, compared to a Background Ratio of 8.4, where only 8.4% of all expressed genes are associated with it. This suggests that the process is overrepresented in the significant genes, indicating its potential biological relevance. The difference between the Gene Ratio and Background Ratio highlights possible enrichment, which may point to the process's role in the condition being studied. The figure highlights processes related to protein synthesis, metabolic activity, cell death regulation, and cellular structure, indicating that these are highly enriched in the dataset.
**Figure S3:** Pairwise similarity matrix of features grouped into 22 clusters (C1–C22). Rows and columns are ordered by cluster membership, with white lines separating clusters. Darker red indicates higher pairwise similarity, while lighter colors indicate lower similarity. Prominent diagonal blocks reflect stronger within‐cluster relationships.
**Figure S4:** (a) Variable importance plot for 20‐top significant genes identified through Elastic Net regularization within the TDEA framework, distinguishing GBM from healthy controls. The analysis was conducted on a training cohort (50 GBM, 191 healthy individuals) from an initial set of 4412 genes. DESeq2 analysis and Elastic Net refinement narrowed the list to 103 highly significant genes, with the top 20 genes displayed based on importance scores. Gray bars (right of the *x*‐axis at 0) represent genes with higher expression in healthy individuals, while red bars (left of the *x*‐axis at 0) indicate elevated expression in GBM patients. Delineated list of the top 20 most crucial genes, including but not limited to: *B2M, CAMK2D, PIK3IP1, LGALS2, UBE2C, CBX7, KALRN, MAOB, FKBP5, CXCR2P1, HSPH1, COL6A3, OAS1, LY86‐AS1, BANK1, SRPR, KSR1, ABCC3, CA1, RP11‐162D16.2* with the complete list of 103 significant genes is provided in Table S6. (b) Confusion matrix from model validation on the test set, demonstrating a high discriminative power with an AUC of 0.99 and a minimal misclassification rate (5%). This highlights the robustness and accuracy of the model in differentiating GBM from healthy controls.
**Figure S5:** Performance of the original 103‐gene signature compared with randomly selected 103‐gene sets. Boxplots and individual points show the distributions of test balanced accuracy (left) and test AUC (right) obtained using random gene sets. The orange horizontal lines indicate the performance of the original signature, which achieved a balanced accuracy of 0.9736 and an AUC of 0.9943, exceeding the corresponding random‐set medians of 0.8449 and 0.9129, respectively (both *p* = 0.0099).

## Data Availability

Data and scripts can be found https://github.com/KrzysztofPastuszak/Platelets‐GBM. All analyses were conducted using R version 4.1.2 or higher.
